# The Complete Inpatient Record Using Comprehensive Electronic Data (CIRCE) project: A team‐based approach to clinically validated, research‐ready electronic health record data

**DOI:** 10.1002/lrh2.10439

**Published:** 2024-06-18

**Authors:** Andrea L. C. Schneider, Jennifer C. Ginestra, Meeta Prasad Kerlin, Michael G. S. Shashaty, Todd A. Miano, Daniel S. Herman, Oscar J. L. Mitchell, Rachel Bennett, Alexander T. Moffett, John Chandler, Atul Kalanuria, Zahra Faraji, Nicholas S. Bishop, Benjamin Schmid, Angela T. Chen, Kathryn H. Bowles, Thomas Joseph, Rachel Kohn, Rachel R. Kelz, George L. Anesi, Monisha Kumar, Ari B. Friedman, Emily Vail, Nuala J. Meyer, Blanca E. Himes, Gary E. Weissman

**Affiliations:** ^1^ Department of Neurology University of Pennsylvania Perelman School of Medicine Philadelphia Pennsylvania USA; ^2^ Department of Biostatistics, Epidemiology and Informatics University of Pennsylvania Perelman School of Medicine Philadelphia Pennsylvania USA; ^3^ Department of Medicine University of Pennsylvania Perelman School of Medicine Philadelphia Pennsylvania USA; ^4^ Palliative and Advanced Illness Research (PAIR) Center University of Pennsylvania Perelman School of Medicine Philadelphia Pennsylvania USA; ^5^ Department of Pathology and Laboratory Medicine University of Pennsylvania Perelman School of Medicine Philadelphia Pennsylvania USA; ^6^ Center for Resuscitation Science University of Pennsylvania Philadelphia Pennsylvania USA; ^7^ Leonard Davis Institute of Health Economics University of Pennsylvania Philadelphia Pennsylvania USA; ^8^ University of Pennsylvania Perelman School of Medicine Philadelphia Pennsylvania USA; ^9^ Department of Health Care Management Wharton School at the University of Pennsylvania Philadelphia Pennsylvania USA; ^10^ Department of Biobehavioral Health Sciences University of Pennsylvania School of Nursing Philadelphia Pennsylvania USA; ^11^ Department of Anesthesia and Critical Care University of Pennsylvania Perelman School of Medicine Philadelphia Pennsylvania USA; ^12^ Department of Surgery University of Pennsylvania Perelman School of Medicine Philadelphia Pennsylvania USA; ^13^ Center for Surgery and Health Economics, Department of Surgery University of Pennsylvania Perelman School of Medicine Philadelphia Pennsylvania USA; ^14^ Department of Emergency Medicine University of Pennsylvania Perelman School of Medicine Philadelphia Pennsylvania USA

**Keywords:** electronic health record (EHR), learning health systems, learning healthcare systems

## Abstract

**Introduction:**

The rapid adoption of electronic health record (EHR) systems has resulted in extensive archives of data relevant to clinical research, hospital operations, and the development of learning health systems. However, EHR data are not frequently available, cleaned, standardized, validated, and ready for use by stakeholders. We describe an in‐progress effort to overcome these challenges with cooperative, systematic data extraction and validation.

**Methods:**

A multi‐disciplinary team of investigators collaborated to create the Complete Inpatient Record Using Comprehensive Electronic Data (CIRCE) Project dataset, which captures EHR data from six hospitals within the University of Pennsylvania Health System. Analysts and clinical researchers jointly iteratively reviewed SQL queries and their output to validate desired data elements. Data from patients aged ≥18 years with at least one encounter at an acute care hospital or hospice occurring since 7/1/2017 were included. The CIRCE Project includes three layers: (1) raw data comprised of direct SQL query output, (2) cleaned data with errors removed, and (3) transformed data with standardized implementations of commonly used case definitions and clinical scores.

**Results:**

Between July 1, 2017 and December 31, 2023, the dataset captured 1 629 920 encounters from 740 035 patients. Most encounters were emergency department only visits (*n* = 965 834, 59.3%), followed by inpatient admissions without an intensive care unit admission (*n* = 518 367, 23.7%). The median age was 46.9 years (25th–75th percentiles = 31.1–64.7) at the time of the first encounter. Most patients were female (*n* = 418 303, 56.5%), a significant proportion were of non‐White race (*n* = 272 018, 36.8%), and 54 625 (7.4%) were of Hispanic/Latino ethnicity.

**Conclusions:**

The CIRCE Project represents a novel cooperative research model to capture clinically validated EHR data from a large diverse academic health system in the greater Philadelphia region and is designed to facilitate collaboration and data sharing to support learning health system activities. Ultimately, these data will be de‐identified and converted to a publicly available resource.

## 1 INTRODUCTION

Over the past two decades, hospitals in the United States have rapidly adopted electronic health record (EHR) systems, with near universal use (>98%) reported by 2018.^1^, ^2^, ^3^ The widespread utilization of EHRs has resulted in extensive digital data archives that hold great promise to support clinical research, hospital operations, and the development of learning health systems.^4^, ^5^ Although EHRs contain rich data, they were not primarily designed for these purposes. As a result, the availability of easily accessible, standardized, and ready‐for‐use EHR data sources has been limited.^6^

Stakeholders, including clinicians, hospital operations and quality officers, and clinical researchers, rely on EHR data to advance their missions and learning health systems benefit from improved access and sharing practices.^7^ Better access to knowledge generated from EHR data will ultimately benefit patients with improved evidence‐based clinical and educational practices and care coordination.^4^, ^5^ There have been several other initiatives to create large datasets for collaborative use from EHR data,^8^, ^9^, ^10^, ^11^, ^12^, ^13^, ^14^ but these initiatives are limited by only including data from one hospital^8^, ^9^, ^10^, ^12^, ^13^, ^14^ and by the inclusion of only intensive care unit (ICU) data.^8^, ^9^, ^11^, ^12^, ^13^, ^14^ Other initiatives have focused on the harmonization of data elements across institutions, including the Observational Medical Outcomes Partnership (OMOP) common data elements and the Patient‐Centered Outcomes Research Institute (PCORI), PCORnet common data model, among others.^15^, ^16^, ^17^ Inspired by the limitations of the relatively small number of other readily available, publicly available EHR‐based data sources,^8^, ^9^, ^10^, ^11^, ^12^, ^13^, ^14^ and by the growing need within our own institution to develop a learning health system, our team sought to draw from the experiences of other initiatives to create an innovative resource at Penn Medicine (comprised of the University of Pennsylvania Health System, the Perelman School of Medicine and School of Nursing at the University of Pennsylvania) to facilitate knowledge creation through the harmonization of data‐focused efforts among a wide range of stakeholders in our academic medical center. We report our process and experiences in hopes this information will serve as a roadmap to other teams building cooperative data resources in their learning health systems.

## 2 RESEARCH INTEREST

Here we describe the rationale, design, data elements, clinical validation, and patient characteristics for the novel Complete Inpatient Record Using Comprehensive Electronic Data (CIRCE) Project. To best align with existing EHR‐based data sources,^8^, ^9^, ^10^, ^11^, ^12^, ^13^, ^14^ we focused our initial efforts on hospital‐based encounter data. The CIRCE Project uses a team‐based, cooperative approach to create a clinically validated, research‐ready dataset comprised of EHR data from all hospital‐based encounters within a large academic health system located in the greater Philadelphia region. The system includes six entities ranging from suburban community hospitals to urban, quaternary referral centers. Philadelphia includes one of the most diverse populations in the country with 57% of the population comprised of women, 40% comprised of individuals of self‐reported Black race and 16% of Hispanic or Latino ethnicity, and a wide range of socioeconomic statuses represented,^18^ which further enhances the potential value of the CIRCE dataset, as many of our patients are underrepresented in existing datasets. Indeed, the CIRCE dataset was created with careful attention to diversity, equity, and inclusion. The overall goal of the CIRCE Project is to promote collaboration, data sharing, harmonization of data elements, and facilitation of access to data for research, quality improvement, and hospital operations, with a public contribution to the open science community. Herein we highlight work completed to date, lessons learned, and future directions for the CIRCE Project as a resource for other teams working to create rich EHR‐based data resources in their own evolving learning health systems.

## 3 METHODS

### 3.1 CIRCE Project investigators

A diverse multi‐disciplinary team of investigators was recruited to develop the CIRCE Project. Investigators contributed a wide breadth of clinical expertise in internal medicine, surgery, anesthesiology, emergency medicine, pulmonary medicine, critical care, neurology, nursing, pharmacy, infectious disease, pathology, and laboratory medicine. The investigative team also represented a broad array of research methods expertise including epidemiology, biostatistics, health services, clinical trials, clinical informatics, business analytics, and data science. Finally, the team also included data analysts with expertise in the health system's underlying EHR data model. Team members identified broad categories of data that would be of interest to research efforts (e.g., laboratory results and vital signs) and grouped them into distinct tables. This grouping created a modular structure for the project to facilitate efficient collaboration among a large group of team members. Each data table was assigned to between one and three clinical faculty members with relevant practice experience to oversee, review, and clinically validate its development in concert with a trained data analyst. For example, the medications table is overseen by several physicians and a practicing clinical pharmacist, and the laboratory/microbiology table is overseen by physicians with expertise in laboratory medicine and pathology, internal medicine, and infectious disease.

### 3.2 CIRCE Project population

The CIRCE Project includes data from six entities within the University of Pennsylvania Health System: (1) The Hospital of the University of Pennsylvania (988‐bed academic quaternary care hospital in Philadelphia, Pennsylvania), (2) The Hospital of the University of Pennsylvania—Cedar Avenue (157‐bed tertiary care hospital in Philadelphia, Pennsylvania), (3) Penn Presbyterian Medical Center (344‐bed academic quaternary care hospital in Philadelphia, Pennsylvania), (4) Pennsylvania Hospital (520‐bed academic tertiary care hospital in Philadelphia, Pennsylvania), (5) Chester County Hospital (329‐bed suburban community hospital in West Chester, Pennsylvania), and (6) Penn Medicine Princeton Health (355‐bed suburban community hospital in Plainsboro Township, New Jersey). Data from all patients aged 18 years or older with at least one emergency department visit, observation stay, inpatient, or hospice admission occurring since July 1, 2017, will be included; the current dataset contains data from July 1, 2017, through December 31, 2023. Patients who opted out of research (per institutional estimates, approximately 13% of patients over the study period opted out of the use of their data for research) or encounters containing information on conditions exempted under Pennsylvania or New Jersey state privacy laws (e.g., human immunodeficiency virus status) were excluded.

The CIRCE Project was approved by the University of Pennsylvania Institutional Review Board (Protocol Number: 853717) and a waiver of consent was granted.

### 3.3 Creation of the CIRCE Project dataset

**FIGURE 1:**
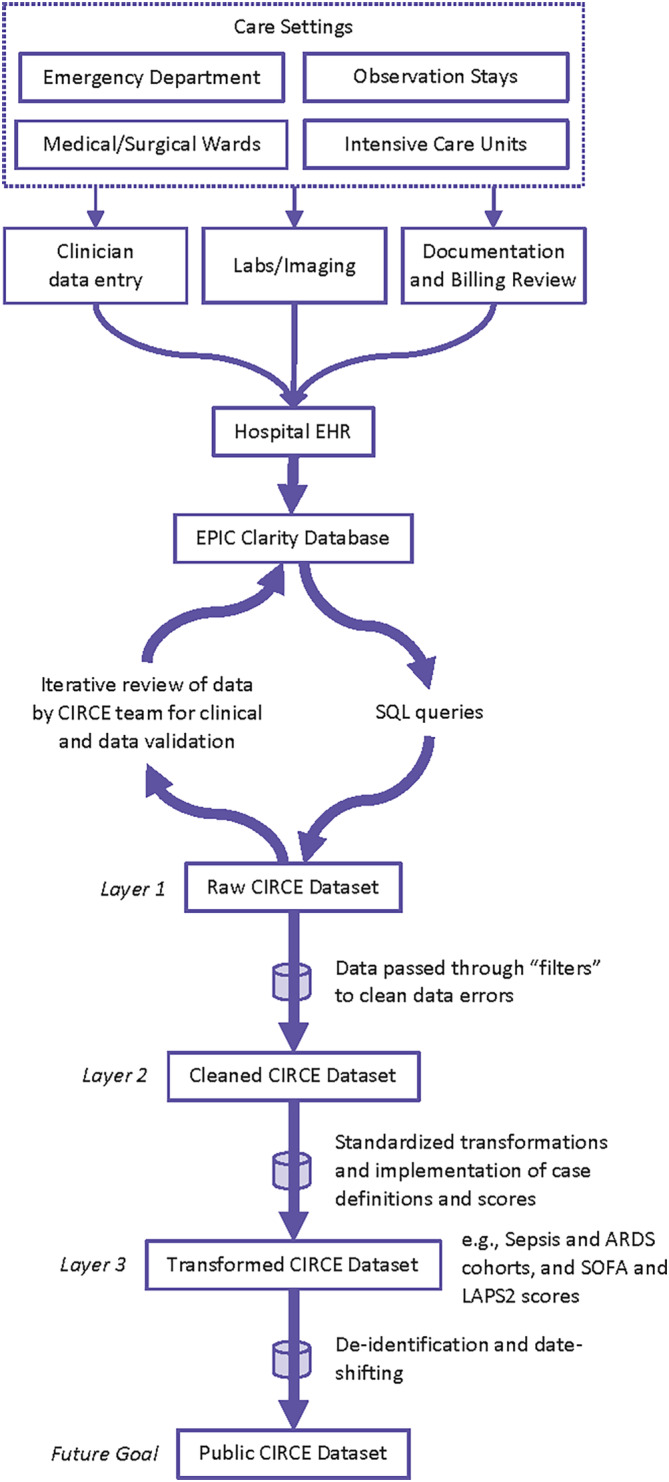
Overview of the Complete Inpatient Record Using Comprehensive Electronic Data (CIRCE) Project. ARDS, acute respiratory distress syndrome; EHR, electronic health record; LAPS2, Laboratory‐based Acute Physiology Score, version 2; SOFA, Sequential Organ Failure Assessment.

An overview of the creation of the CIRCE Project dataset is depicted in Figure 1. The source data for the CIRCE Project are generated during routine clinical care in the health system EHR (Epic). They are archived within the secure University of Pennsylvania Health System Epic Clarity Database (a Microsoft SQL Server Database). The multi‐disciplinary team of CIRCE Project investigators collaborated to select data fields to query that are relevant to clinical research, quality improvement, and clinical operations. We undertook a structured and iterative validation process for each data table. First, the clinical faculty lead(s) for each table met with the team's data scientist to develop a list of candidate fields and their clinical context. These meetings often involved real‐time data extraction using SQL queries on a shared screen in a virtual meeting to confirm the content associated with each field. Then a few months of data were extracted for the table and underwent iterative review by the clinical faculty leads for clinical relevance, missingness, errors, and duplicate records. The data scientist updated these tables iteratively based on this feedback, sometimes producing over a dozen versions of the query. In the final step, each table lead was asked to fill out a structured survey instrument in RedCAP to document any final feedback about content, errors, clinical relevance, or other important fields that were missing were then incorporated into the query. A final review of the queries and data extracted over several years was reviewed by the study team. Table leads were also encouraged to view and provide feedback for other tables not directly supervised by them in order to increase the robustness of the review process and ensure data elements aligned with stakeholder needs.

This review and validation process is being performed iteratively to create layers of the CIRCE dataset, with the first layer being the raw CIRCE dataset (i.e., *Layer 1* data in Figure 1). The dataset resulting after passing through “filters” (e.g., correcting data entry errors, formatting errors, and typos, as well as evaluation of the distribution of variables and investigation into outliers with clinical validation) is the cleaned CIRCE dataset (i.e., *Layer 2* data in Figure 1). The dataset was created after undergoing standard transformations and implementation of widely used case definitions and scores (e.g., creation of derived variables for race and ethnicity, identification of clinical cohorts including sepsis, acute respiratory failure, and stroke, among others, and calculation of in‐hospital mortality risk scores, such as the Sequential Organ Failure Assessment (SOFA) score^19^ and Laboratory‐based Acute Physiology Score, version 2 (LAPS2)),^20^ is the transformed CIRCE dataset (*Layer 3* data in Figure 1). The transformed CIRCE dataset also contains limited identifiable data that are appropriate for work needing to link to external datasets (e.g., neighborhood‐level socioeconomic, environmental data, administrative data) and for temporally sensitive work (e.g., related to coronavirus disease [COVID‐19]). Lastly, in a future CIRCE Project effort, the data will be completely de‐identified by removing the 18 identifiers described in the Health Insurance Portability and Accountability Act (HIPAA) and implementing a random date shift at the encounter level (to preserve within‐encounter temporal relationships) to create a free and publicly available version of the CIRCE dataset. The CIRCE Project dataset will include data beginning in July 2017, aligned with our health system's adoption of the current EHR system for inpatient use, and is expected to be updated monthly.

### 3.4 CIRCE Project dataset access

A broad range of stakeholders will have access to the CIRCE dataset with restrictions according to the degree of protected health information included in the data. Access to the raw, cleaned, and transformed CIRCE datasets (which contain identifiable data) will be granted for individuals with Penn employment affiliations or with data use agreements for those projects with approval from an institutional review board. In the future, access to the fully de‐identified and publicly available CIRCE dataset will require documented training in the protection of human subjects (which includes HIPAA) and a signed data use agreement. All data tables will be provided as comma‐separated value (CSV) files. Shared coding resources and codebooks containing detailed descriptions of how each derived variable was defined in the clinical validation and data cleaning process will be made publicly available on GitHub.

## 4 RESULTS

### 4.1 Classes of data available in the CIRCE Project dataset

**FIGURE 2:**
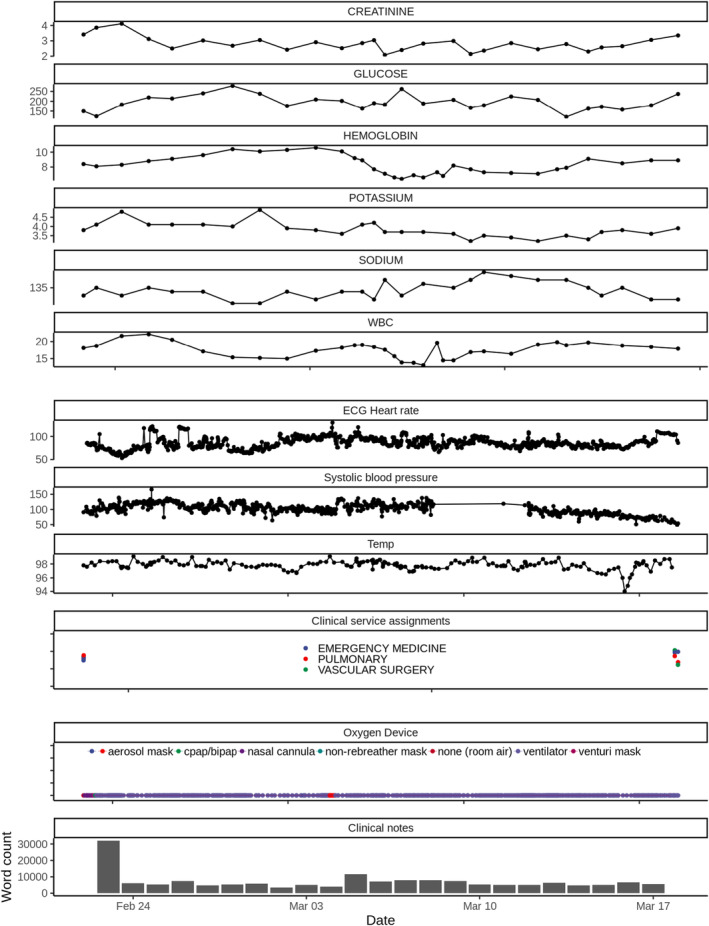
Visualization of data over time for a single patient encounter as an example of data sources available. BiPAP, bilevel positive airway pressure; CPAP, continuous positive airway pressure; ECG, electrocardiogram; Temp, temperature; WBC, white blood cell count.

A total of 18 data tables containing data from July 1, 2017 to December 31, 2023 comprises the initial version of the CIRCE Project dataset (see Table 1 for descriptions of available classes of data). All data tables are relational, including keys (such as the enterprise master person index [EMPI]) that enable users to link data across tables by patient and encounter across included hospitals. Figure 2 shows a visualization of data over time for a single patient admission, highlighting the rich array of data sources available, including laboratory values (creatinine, glucose, hemoglobin, potassium, sodium, and white blood count [WBC]), vital signs (heart rate, systolic blood pressure, and temperature), clinical service assignments, oxygen device, and word counts of clinical notes.

**TABLE 1:** Description of classes of data available in the CIRCE Project.

Class of Data	Description
Patient	Patient demographic information, including each individual's enterprise master person index (EMPI), age, race, ethnicity, preferred language, insurance, gender, and sex assigned at birth, including changes to race, ethnicity, and gender over time.
Encounters	Detailed information about every hospital‐based encounter, including emergency, observation, and acute inpatient visits, including presentation, admission, discharge timestamps, admitting source, and discharge disposition.
Administrative	Administrative and billing data, including Major Diagnostic Category (MDC) codes, Current Procedural Terminology (CPT) codes, Diagnosis‐Related Group (DRG) codes, and International Classification of Disease, Tenth Revision (ICD‐10) codes with present‐on‐admission indicators.
Location	Locations of care delivery from presentation through discharge, including location names, location type (e.g., ward, intensive care unit, operating room, radiology), and timestamps of arrival and departure from each patient care location.
Team coverage	Covering service line and team (e.g., “MICU team A”), including roles of bedside providers and nurses, attending physicians, and other clinical team members, and coverage time periods for each team and team member.
Laboratory/microbiology	All laboratory test results, including microbiology (e.g., cultures, respiratory viral panels, bacterial, viral, fungal qualitative and quantitative serologies, and polymerase chain reaction tests), associated drug dosing, safety monitoring, and infection and inflammation assessment studies. All laboratory results are mapped to LOINC.
Vital signs	Nurse‐verified flowsheet values including temperature, heart rate, blood pressure, respiratory rate, oxygen saturation, oxygen delivery device, oxygen flow rate, and the fraction of inspired oxygen with associated timestamps.
Medications	Medication order, pharmacy dispensing information, and medication administration data, including medication name, dosage, concentration, frequency, route of administration, timestamps of order entry, pharmacy dispensing, and medication administration. All medications are mapped to RxNorm.
Immunizations	Vaccination data for influenza, pneumococcal pneumonia, and SARS‐CoV‐2/COVID‐19, including vaccine product (including booster designations) and manufacturer, administration date, dosage, and administration site.
Input and output	Recorded input and output, including volume administered as medications or intravenous fluids, nutritional intake by route, and output volume by source.
Code status	Orders reflecting full code, limitations on life‐sustaining therapy, and comfort measures with associated timestamps.
Orders	Non‐medication and non‐laboratory orders, including diagnostic imaging, consult requests, diet type, isolation status, and non‐surgical procedures (e.g., thoracentesis, endotracheal intubation, and radiology‐guided biopsy).
Clinical notes	Free text of all notes, including history and physical notes, progress notes, consult notes, discharge summaries, nursing notes, notes from other allied health team members, and information on timestamps indicating when notes were signed with potential identifying information such as phone numbers removed through automated means.
ICU Flowsheets	Data from structured fields from flowsheets used primarily in intensive care units, measured and recorded either automatically or by nurses, respiratory therapists, or clinical perfusionists, including settings and readings from respiratory, cardiac, and neurological monitoring and support devices.
Nursing flowsheets	Nurse‐documented assessments including daily shift assessments, fall risk, pressure ulcer risk, activities of daily living, delirium, and wounds.
Anesthesia flowsheets	Data collected during anesthesia care, including vital signs, medications (intravenous and inhaled), fluids, and blood products administered by anesthesia providers, inputs and outputs, and event timestamps and notes for anesthesia procedures.
Surgery	Information on surgical procedures, including CPT codes, elective versus emergent status, surgical site, devices, hardware, and event timestamps.
Emergency department (ED)	Aspects of ED care, including reason for visit, ED team shift schedule, means of arrival, pre‐arrival notification, admitting/provisional diagnosis, and disposition (admission, discharge, or transfer).

### 4.2 Characteristics of the CIRCE Project dataset

Table 2 shows both encounter‐level and patient‐level characteristics overall and stratified by year to provide a description of key features in the dataset. The dataset captured 1 629 920 encounters from 740 035 unique patients. Among these patients, the median age was 46.9 years (25th–75th percentiles: 31.1–64.7) at the time of the first encounter in the study period. The majority of patients were of female sex (56.5%), 36.8% of participants were of non‐White race, and 7.4% were of Hispanic or Latino ethnicity. The greatest proportion of patients were employed full‐time (38.4%), while 21.8% were retired, and 16.8% were not employed. The most common type of health insurance was managed care (32.6%), followed by managed Medicaid (17.0%), and Medicare (16.0%).

**TABLE 2:** Encounter‐ and patient‐level characteristics, overall and stratified by year (July 1, 2017–December 31, 2023).

	Overall	2017^a^	2018	2019	2020	2021	2022	2023
Encounter‐level characteristics								
Total number of encounters	1 629 920	101 853	224 458	251 313	216 684	264 967	278 650	291 995
Type of encounter, *n* (%)								
Emergency department only	965 834 (59.3)	56 289 (55.3)	126 547 (56.4)	143 806 (57.2)	122 161 (56.4)	158 786 (59.9)	172 707 (62.0)	185 538 (63.5)
Observation	107 238 (6.6)	6411 (6.3)	15 784 (7.0)	17 787 (7.1)	13 560 (6.3)	17 146 (6.5)	17 642 (6.3)	18 908 (6.5)
Inpatient admission with intensive care unit (ICU) admission	35 883 (1.6)	2730 (1.9)	5597 (1.8)	5746 (1.7)	5241 (1.8)	5513 (1.6)	5504 (1.5)	5552 (1.5)
Inpatient admission without ICU admission	518 367 (23.7)	36 309 (25.8)	76 235 (24.9)	83 630 (24.5)	75 332 (25.3)	83 080 (25.3)	82 356 (22.5)	81 425 (21.5)
Inpatient hospice	2598 (0.2)	114 (0.1)	295 (0.1)	344 (0.1)	390 (0.2)	442 (0.2)	441 (0.2)	572 (0.2)
Primary encounter Major Diagnostic Category (MDC)^b^, *n* (%)								
Diseases and disorders of the circulatory system	177 673 (17.5)	12 645 (20.4)	27 220 (19.6)	29 937 (17.4)	24 842 (16.1)	28 017 (17.1)	27 745 (17.0)	27 267 (16.9)
Diseases and disorders of the musculoskeletal system and connective tissue	115 227 (11.4)	9370 (15.1)	18 531 (13.3)	21 600 (12.6)	17 303 (11.2)	17 087 (10.4)	15 569 (9.6)	15 767 (9.8)
Diseases and disorders of blood, blood‐forming organs, and immunologic disorders	100 996 (10.0)	1076 (1.7)	6680 (4.8)	18 500 (10.8)	17 932 (11.6)	19 149 (11.7)	19 404 (11.9)	18 255 (11.3)
Diseases and disorders of the nervous system	94 805 (9.3)	6906 (11.2)	14 449 (10.4)	15 216 (8.9)	13 555 (8.8)	14 931 (9.1)	14 961 (9.2)	14 787 (9.2)
Diseases and disorders of the digestive system	88 759 (8.7)	5879 (9.5)	12 990 (9.3)	14 898 (8.7)	13 092 (8.5)	14 573 (8.9)	13 583 (8.3)	13 744 (8.5)
Diseases and disorders of the respiratory system	84 428 (8.3)	4478 (7.2)	11 303 (8.1)	12 306 (7.2)	13 738 (8.9)	14 341 (8.7)	14 726 (9.0)	13 536 (8.4)
Other^c^	352 596 (34.8)	21 523 (34.8)	47 771 (34.4)	59 317 (34.5)	53 463 (34.7)	55 973 (34.1)	56 880 (34.9)	57 669 (35.8)
Length of encounter stay, median [25th–75th percentiles]								
Emergency department only (hours)	3.0 [1.8–4.7]	3.2 [1.9–4.9]	3.1 [1.9–4.8]	3.0 [1.8–4.6]	2.9 [1.7–4.5]	2.9 [1.6–4.6]	2.9 [1.7–4.6]	3.1 [1.9–4.8]
Observation (days)	1.2 [0.9–1.8]	1.1 [0.8–1.6]	1.1 [0.9–1.6]	1.1 [0.9–1.6]	1.1 [0.9–1.7]	1.2 [0.9–1.8]	1.2 [0.9–1.9]	1.3 [0.9–2.0]
Inpatient admission (with or without ICU admission) (days)	3.6 [2.3–6.3]	3.4 [2.3–6.0]	3.4 [2.3–6.1]	3.4 [2.3–6.1]	3.4 [2.2–6.2]	3.8 [2.3–6.6]	3.8 [2.3–6.8]	3.8 [2.3–6.6]
Inpatient hospice (days)	1.3 [0.6–2.9]	1.0 [0.7–2.0]	1.2 [0.7–2.8]	1.4 [0.6–3.0]	1.3 [0.6–2.9]	1.2 [0.6–2.6]	1.4 [0.5–2.9]	1.4 [0.6–3.0]
In‐hospital mortality, *n* (%)	16 069 (1.0)	844 (0.8)	2068 (0.9)	2200 (0.9)	2524 (1.2)	2747 (1.0)	2860 (1.0)	2826 (1.0)
Patient‐level characteristics^d^								
Total number of unique patients	740 035	84 983	133 164	123 469	91 968	104 871	102 241	99 339
Age (years), median [25th–75th percentiles]	46.9 [31.1–64.7]	47.7 [30.8–64.4]	47.5 [31.1–64.7]	47.5 [31.2–65.0]	46.6 [31.3–64.1]	46.0 [31.0–64.1]	46.4 [31.0–65.0]	46.0 [30.9–65.2]
Sex, *n* (%)								
Female	418 303 (56.5)	51 197 (60.2)	78 089 (58.6)	70 910 (57.4)	50 906 (55.4)	57 285 (54.6)	55 928 (54.7)	53 988 (54.3)
Male	321 667 (43.5)	33 785 (39.8)	55 074 (41.4)	52 557 (42.6)	41 057 (44.6)	47 576 (45.4)	46 300 (45.3)	45 318 (45.6)
Other/not reported	65 (<0.1)	1 (<0.1)	1 (<0.1)	2 (<0.1)	5 (<0.1)	10 (<0.1)	13 (<0.1)	28 (<0.1)
Race, *n* (%)								
Asian	37 438 (5.1)	2372 (2.8)	5618 (4.2)	6293 (5.1)	4794 (5.2)	5676 (5.4)	6275 (6.1)	6410 (6.5)
Black or African American	233 063 (31.5)	35 785 (42.1)	43 677 (32.8)	35 682 (28.9)	28 141 (30.6)	33 684 (32.1)	29 592 (28.9)	26 502 (26.7)
White	385 934 (52.2)	40 557 (47.7)	70 740 (53.1)	67 926 (55.0)	48 307 (52.5)	52 837 (50.4)	53 071 (51.9)	52 496 (52.8)
Other^e^	38 955 (5.3)	2645 (3.1)	5350 (4.0)	5176 (4.2)	3960 (4.3)	4884 (4.7)	6772 (6.6)	10 168 (10.2)
Not reported	44 645 (6.0)	3624 (4.3)	7779 (5.8)	8392 (6.8)	6766 (7.4)	7790 (7.4)	6531 (6.4)	3763 (3.8)
Ethnicity, *n* (%)								
Hispanic or Latino	54 625 (7.4)	4441 (5.2)	8612 (6.5)	8521 (6.9)	6968 (7.6)	7850 (7.5)	8742 (8.6)	9491 (9.6)
Not Hispanic or Latino	675 538 (91.3)	79 970 (94.1)	123 025 (92.4)	113 162 (91.7)	83 666 (91.0)	95 435 (91.0)	92 032 (90.0)	88 248 (88.8)
Not reported	9872 (1.3)	572 (0.7)	1527 (1.1)	1786 (1.4)	1333 (1.4)	1587 (1.5)	1467 (1.4)	1600 (1.6)
Employment status, *n* (%)								
Full‐time	284 131 (38.4)	28 995 (34.1)	50 141 (37.7)	48 848 (39.6)	36 980 (40.2)	41 335 (39.4)	39 514 (38.6)	38 318 (38.6)
Part‐time	31 733 (4.3)	3983 (4.7)	6161 (4.6)	5413 (4.4)	3942 (4.3)	4389 (4.2)	4168 (4.1)	3677 (3.7)
Student	33 102 (4.5)	2587 (3.0)	5709 (4.3)	6110 (4.9)	3592 (3.9)	4741 (4.5)	5127 (5.0)	5236 (3.7)
Retired	161 616 (21.8)	20 754 (24.4)	31 549 (23.7)	28 493 (23.1)	19 446 (21.1)	21 348 (20.4)	20 730 (20.3)	19 296 (19.4)
Not employed	123 958 (16.8)	14 908 (17.5)	21 675 (16.3)	18 633 (15.1)	16 128 (17.5)	18 604 (17.7)	17 573 (17.2)	16 437 (16.5)
Disabled	37 005 (5.0)	8217 (9.7)	8305 (6.2)	6023 (4.9)	3964 (4.3)	4263 (4.1)	3515 (3.4)	2718 (2.7)
Not reported	68 490 (9.3)	5539 (6.5)	9624 (7.2)	9949 (8.1)	7916 (8.6)	10 191 (9.7)	11 614 (11.4)	13 657 (13.7)
Insurance, *n* (%)								
Commercial	84 105 (11.4)	7481 (8.8)	14 078 (10.6)	14 136 (11.4)	10 893 (11.8)	12 711 (12.1)	12 608 (12.3)	12 198 (12.3)
Medicare	118 509 (16.0)	15 919 (18.7)	23 966 (18.0)	21 605 (17.5)	14 211 (15.5)	15 219 (14.5)	14 287 (14.0)	13 302 (13.4)
Medicaid	12 424 (1.7)	1286 (1.5)	1948 (1.5)	1711 (1.4)	1562 (1.7)	2027 (1.9)	2061 (2.0)	1829 (1.8)
Managed Medicare	78 637 (10.6)	11 745 (13.8)	15 096 (11.3)	12 680 (10.3)	9215 (10.0)	10 407 (9.9)	10 097 (9.9)	9397 (9.5)
Managed Medicaid	125 805 (17.0)	19 134 (22.5)	22 648 (17.0)	18 182 (14.7)	15 115 (16.4)	18 573 (17.7)	16 721 (16.4)	15 432 (15.5)
Managed care	241 596 (32.6)	22 139 (26.1)	42 352 (31.8)	42 620 (34.5)	31 653 (34.4)	34 675 (33.1)	34 088 (33.3)	34 069 (34.3)
Self‐Pay	78 061 (10.5)	7202 (8.5)	12 834 (9.6)	12 317 (10.0)	9198 (10.0)	11 151 (10.6)	12 286 (12.0)	13 073 (13.2)
Other/unknown	898 (0.1)	77 (0.1)	242 (0.2)	218 (0.1)	121 (0.1)	108 (0.1)	93 (0.1)	39 (<0.1)

^a^ July 1, 2017 through December 31, 2023.

^b^ Among a subset of encounters eligible for MDC classification (overall *N* = 1 014 489, 2017 *n* = 61 877, 2018 *n* = 138 944, 2019 *n* = 171 774, 2020 *n* = 153 925, 2021 *n* = 164 071, 2022 *n* = 162 868, and 2023 *n* = 161 025).

^c^ Other primary encounter Major Diagnostic Category (MDC) includes alcohol/drug use or induced mental disorders; burns; ear, nose, mouth, and throat; endocrine, nutritional, and metabolic system; eye; factors influencing health status; female reproductive system; hepatobiliary system and pancreas; human immunodeficiency virus infection; infectious and parasitic diseases; injuries, poison and toxic effect of drugs; kidney and urinary tract; male reproductive system; mental diseases and disorders; multiple significant trauma; myeloproliferative disorders; pregnancy, childbirth, and puerperium; and skin, subcutaneous tissue, and breast.

^d^ Patient‐level data from the first encounter during the study period.

^e^ Other race includes American Indian, Alaskan Native, Asian, East Indian, Native Hawaiian, Pacific Islander, and not reported.

The number of encounters per year has increased over time, with a decrease from 2019 to 2020 in the setting of the COVID‐19 pandemic. In total, there were 1 354 434 emergency department encounters and 18.7% of these resulted in inpatient admission. Overall, emergency department only encounters represented the majority of encounters (*n* = 965 834, 59.3% of all encounters), followed by inpatient admissions without ICU admission (*n* = 518 367, 23.7% of all encounters). A subset of inpatient admissions included ICU admission (*n* = 35 883, 1.6% of all encounters). During the study period, the overall median length of inpatient stay (with or without ICU admission) was 3.6 (25th–75th percentiles: 0.2.3–6.3) days. A total of 16 069 (1.0%) encounters resulted in in‐hospital mortality. Among the subset of encounters that were assigned a Diagnosis‐Related Group (DRG) code, thus enabling mapping to a Major Diagnostic Category (MDC) coding (*n* = 1 014 489), the most common primary encounter MDCs were diseases and disorders of the circulatory system (17.5%), diseases and disorders of the musculoskeletal system and connective tissue (11.4%), and diseases and disorders of blood, blood‐forming organs, and immunologic disorders (10.0%), followed by diseases and disorders of the nervous (9.3%), digestive (8.7%), and respiratory (8.3%), systems. In total, the dataset captures 309 782 surgical procedures, with the most common being cesarean deliveries (*n* = 25 721), followed by total knee arthroplasties (*n* = 7821).

## 5 DISCUSSION

The CIRCE Project is creating a novel ready‐for‐use dataset comprised of clinically validated EHR data from six entities within a large and diverse academic health system in the greater Philadelphia region. Using a highly collaborative and iterative approach to the clinical validation of the data elements, the CIRCE Project dataset was developed to promote research and collaboration among stakeholders within Penn Medicine and from other institutions. A close working relationship among clinicians, researchers, and data analysts with a shared interest in high‐quality EHR data provides the foundation for this work. A rigorous de‐identification process will enable future public sharing of the CIRCE dataset.

The CIRCE Project was inspired by other initiatives that have created large datasets for collaborative use from EHR data but differs from those efforts in several important ways.^8^, ^9^, ^10^, ^11^, ^12^, ^13^, ^14^ First, most other efforts have been limited to the creation of ICU‐focused datasets,^8^, ^9^, ^11^, ^12^, ^13^, ^14^ with one (the Medical Information Mart for Intensive Care‐IV [MIMIC‐IV]) also recently incorporating emergency department and other inpatient data.^10^ Second, most existing datasets have been limited to one hospital,^8^, ^9^, ^10^, ^12^, ^13^, ^14^ although one (the eICU Collaborative Research Database) combines ICU data from 208 hospitals.^11^ Third, the CIRCE Project data maps to standard ontologies such as RxNorm for medication data and LOINC for laboratory data, which facilitates standardization. Further, when multiple LOINC or RxNorm codes appeared for the same test or medication, we aggregated them into a single field separated by semicolons. This approach permits the user of the data to decide which codes are relevant to their particular research question while not abandoning any of the relevant information contained in those codes. Fourth, the CIRCE Project dataset includes granular information about surgical cases associated with inpatient encounters, including associated CPT codes, primary and secondary operators, timestamps related to flow through the peri‐operative holding areas, and granular data about volatile anesthetic use, ventilator settings, and sedative medications during procedures. The CIRCE Project expands upon the strengths of these existing initiatives^8^, ^9^, ^10^, ^11^, ^12^, ^13^, ^14^ to create a large, multi‐center dataset containing information relating to patients receiving medical care across six distinct healthcare settings, ranging from the emergency department to the ICU to inpatient hospice. Indeed, the inclusion of the EMPI not only allows for linkage across included hospitals but also to other resources including the Penn Medicine Biobank.^21^ Moreover, the wide breadth of expertise of the CIRCE investigators enables the collection of a complete inpatient record with clinical relevance and robust construct validity across numerous domains, including but not limited to medical, surgical, nursing, pharmacy, and laboratory care processes. The iterative clinical validation process is a strength over other propriety data platforms.

Another important strength of the CIRCE Project is the attention to curating data in a manner that is focused on diversity, equity, and inclusion. Indeed, the diversity of the patients who receive care at Penn Medicine is a distinct strength of the CIRCE Project and may help us to conduct health disparities research in the futures. Additionally, the patient demographic variables include all available sex, gender, race, ethnicity, preferred language, insurance, as well as other socioeconomic factors. This is especially important for patients who identify as multiple race and ethnicity categories and whose additional race and ethnicity identifications may not always be included in existing datasets. However, it is important to note that race and ethnicity data in EHRs often comes from multiple sources and cannot be assumed to represent an individual patient's self‐identification. The data will also include changes to demographic categories and their timestamps. This is particularly important for patients whose identification with respect to gender, race, ethnicity, or other categories may change over time. In addition, the transformed CIRCE dataset (*Layer 3*) includes geographic information to facilitate merging with external data sources that contain neighborhood‐level information about social, environmental, and other relevant exposures.^22^, ^23^, ^24^, ^25^ These strengths of the CIRCE Project will facilitate research focused on historically marginalized groups that are critical to advancing diversity, equity, and inclusion efforts through high‐quality science relying on EHR‐based data.

### 5.1 Lessons learned

The experiences of the CIRCE Project team members yield several lessons for multidisciplinary groups of stakeholders at other institutions who are developing comprehensive EHR‐based datasets to support learning health system activities. First, an umbrella institutional review board (IRB) approval is an effective and efficient way to protect humans participating in research through routine data collection for a large number of investigators contributing to integrated projects. Although upfront time and costs in preparing a joint IRB and ongoing time and costs related to managing modifications do require investment, the total time and cost savings compared to dozens of separate IRB protocols are large for both investigators and the local IRB.

Second, the conduct of the CIRCE Project bridged disciplines in such a way that it engendered camaraderie, facilitated new working relationships and collaborations, and identified research, quality improvement, and operational opportunities among many stakeholders across departments and between the university and health system. The creation of this large‐scale dataset yields a rich resource far beyond any individual's investment that is efficient and promotes work‐related satisfaction in a team environment. Moreover, the multidisciplinary team of CIRCE Project investigators enabled the database to capture a more complete view of care delivery across intersecting and disparate domains of inpatient care than would have been possible with more a traditionally siloed team of investigators.

Third, the construction of a dataset like this is itself an opportunity to facilitate the growth and development of a learning health system.^4^, ^5^ As we described the eventual resource that will emerge from the CIRCE Project to local collaborators, more and more stakeholders realized its potential benefits, which in turn led to snowballing support. The ensuing conversations around roles, access, and support have brought different stakeholders in our university and health system closer in aligning work and understanding overlap across divergent efforts and missions.

Fourth, federal and state laws around data‐sharing, privacy, and accountability should inform large‐scale data aggregation efforts in a learning health system. Our effort required close collaboration among the CIRCE Project team members, IRB representatives, leaders in the Office of Clinical Research (OCR), the Chief Privacy Officer, and those from the Data Analytics Core. Specifically, our team sought clear guidance not only on which patients and encounters should be excluded from the data extract but also on how to accurately operationalize those definitions using appropriate flags and other codes in the EHR data. This implementation was highly specific to our health system as it reflected local health system policies (e.g., patient opt‐out preferences) and state laws from Pennsylvania and New Jersey governing patient privacy. These data protection and privacy steps have been necessary for our early work in pulling and sharing data internally, not just for the future planned public sharing of a de‐identified version of the CIRCE dataset. We suggest close consultation with the relevant experts to clarify these restrictions, and protections, and their implementation, as early as possible.

### 5.2 Future directions

The CIRCE Project data tables will continue to be iteratively revised in response to ongoing clinical validation and testing by team members. Although the current iteration of the CIRCE Project incorporates LOINC and RxNorm codes for all tests and medications, for administrative and logistical reasons, our effort was unable to utilize the OMOP common data model at the time CIRCE was created. In future work, we will consider building on and/or integrating the OMOP ontology elements with the CIRCE Project. In addition, future iterations will incorporate additional waveform data captured continuously and automatically by medical support devices as well as incorporate the Food and Drug Administration's unique device identification system to facilitate the study of the safety and effectiveness of medical devices.^26^ Future iterations will also incorporate imaging data (e.g., both images and radiology reports) and pathology report data. The addition of radiology and pathology report data in addition to the narrative clinical notes will enable end users to integrate natural language processing resources to answer questions of interest. Another area of future interest includes expanding the existing patient encounter classes to include Penn Medicine at home (home care services), infusion clinic, and outpatient data. However, it is important to note that the current CIRCE Project data does include diagnoses from home care, infusion clinic, and outpatient clinic as these are captured in the problem list that spans inpatient and outpatient data and are available in the current “administrative” data table. Future efforts will also be focused on linkage with longitudinal administrative claims data using privacy‐preserving record linkage with tokenization in order to capture health outcomes occurring outside of the University of Pennsylvania Health System.

## 6 CONCLUSIONS

In this paper, we described the collaborative processes that have been implemented to develop and clinically validate the CIRCE Project dataset and highlighted key experiences and lessons learned along the way. The CIRCE Project dataset is an example of how EHR data can be curated in a manner that addresses the privacy, ethical, and legal concerns of multiple stakeholders to support diverse learning health system activities.

## FUNDING INFORMATION

Support for the CIRCE Project was provided by Penn Medicine. Dr. Schneider reports NIH/NINDS funding (K23NS123340) and Department of Defense funding (W81XWH‐21‐1‐0590 and HT9425‐23‐1‐0981). Dr. Mitchell receives funding from the Zoll Foundation. Dr. Moffett was supported by NHLBI F32HL167456. Dr. Joseph was supported by NIH/NIGMS K08GM139031. Dr. Kohn was supported by NIH/NHLBI K23 HL146894. Dr. Kelz was supported by NIH/NIA R01AG060612, NIH/NIMHD R01MD016088, and the University of Pennsylvania Leonard Davis Institute. Dr. Anesi reports research funding from the National Institutes of Health (K23HL161353), the CDC Foundation, the Society of Critical Care Medicine (SCCM), and the University of Pennsylvania Perelman School of Medicine Thomas B. McCabe and Jeannette E. Laws McCabe Fund. Dr. Vail was supported by AHRQ 5K12HS026372. Dr. Meyer was supported by NIH/NHLBI R35HL161196 and U01HL168419. Dr. Himes was supported by NIH/NHLBI R01HL133433 and NIH/NIEHS P30ES013508. The content is solely the responsibility of the authors and does not necessarily represent the official views of the National Institutes of Health or other funding agencies.

## CONFLICT OF INTEREST STATEMENT

Dr. Schneider reports serving as an Associate Editor for the journal *Neurology* from the American Academy of Neurology. Dr. Anesi reports payments for authoring chapters for UpToDate and expert witness consulting, including involving COVID‐19, and reports that his spouse is employed by the U.S. Food and Drug Administration (FDA).
